# Comparison of different cardiovascular risk tools used in HIV patient cohorts in sub-Saharan Africa; do we need to include laboratory tests?

**DOI:** 10.1371/journal.pone.0243552

**Published:** 2021-01-28

**Authors:** Frank Mubiru, Barbara Castelnuovo, Steven J. Reynolds, Agnes Kiragga, Harriet Tibakabikoba, Noela Clara Owarwo, Andrew Kambugu, Mohammed Lamorde, Rosalind Parkes-Ratanshi

**Affiliations:** 1 Infectious Disease Institute, Makerere University, Kampala, Uganda; 2 Division of Intramural Research, National Institute of Allergy and Infectious Diseases, National Institutes of Health, Bethesda, Maryland, United States of America; 3 Johns Hopkins University School of Medicine, Baltimore, Maryland, United States of America; 4 University of Cambridge, Institute of Public Health, Forvie Site, Cambridge, United kingdom; University of Ghana College of Health Sciences, GHANA

## Abstract

**Introduction:**

Cardiovascular disease (CVD) is the leading cause of death globally, representing 31% of all global deaths. HIV and long term anti-retroviral therapy (ART) are risk factors for development of CVD in populations of people living with HIV (PLHIV). CVD risk assessment tools are currently being applied to SSA populations, but there are questions about accuracy as well as implementation challenges of these tools in lower resource setting populations. We aimed to assess the level of agreement between the various cardiovascular screening tools (Data collection on Adverse effects of anti-HIV Drugs (D:A:D), Framingham risk score, WHO risk score and The Atherosclerotic Cardiovascular Disease Score) when applied to an HIV ART experienced population in Sub-Saharan Africa.

**Methods:**

This study was undertaken in an Anti-Retroviral Long Term (ALT) Cohort of 1000 PLHIV in care who have been on ART for at least 10 years in urban Uganda. A systematic review was undertaken to find the most frequently used screening tools from SSA PLHIV populations; these were applied to the ALT cohort. Levels of agreement between the resulting scores (those including lipids and non-lipids based, as well as HIV-specific and non-HIV specific) as applied to our cohort were compared. Prevalence Bias Adjusted Kappa was used to evaluate agreement between tools.

**Results:**

Overall, PLHIV in ALT cohort had a median score of 1.1–1.4% risk of a CVD event over 5 years and 1.7–2.5% risk of a CVD event over 10 years. There was no statistical difference in the risk scores obtained for this population when comparing the different tools, including comparisons of those with lipids and non-lipids, and HIV specific vs non-HIV specific.

**Conclusion:**

The various tools yielded similar results, but those not including lipids are more feasible to apply in our setting. Long-term cohorts of PLHIV in SSA should in future provide longitudinal data to evaluate existing CVD risk prediction tools for these populations. Inclusion of HIV and ART history factors to existing scoring systems may improve accuracy without adding the expense and technical difficulty of lipid testing.

## Introduction

Cardiovascular disease (CVD) is the leading cause of death globally. An estimated 17.9 million people died from CVDs in 2016, representing 31% of all global deaths. Of these deaths, 85% are due to Myocardial Infarction (MI) and Cerebrovascular Accidents (CVA). In Uganda specifically, a recent survey on Non Communicable Diseases (NCDs) by World Health Organisation (WHO) (2016), revealed that 10.5% of adults between 40–69 years have a 30% chance of developing CVDs in the next 10 years [1]. However, unlike in high-income settings, screening for cardio-vascular disease is not routinely undertaken.

The HIV infected population is estimated to be at 1.5–2 fold risk of death from CVD’s as compared to the HIV negative population [2]. The likely pathogenic mechanisms are increased vascular inflammation due to HIV virus circulation. However, there is also a risk attributed to anti-retroviral (ARV) drugs, in part due to increased lipids, with some ARVs including abacavir and protease inhibitors having stronger CVD risk as compared to other drugs [3]. Access to anti-retroviral treatment (ART) has dramatically reduced mortality in people living with HIV (PLHIV) in sub Saharan Africa (SSA) [4,5], but the experience with ART in SSA is shorter (10–15 years) as compared to over 30 years’ experience in high income settings [6].

CVD screening tools such as the Framingham prediction model are based upon data collected from large general population cohorts from high income settings [7]. These include factors such as age, sex, family history, weight, smoking, drug history and previous event history. However, they also rely on availability of comprehensive laboratory test results such as lipid profiles that are not widely available and are very costly in SSA. To counter this, the WHO and others have devised screening tools that do not rely on inclusion of laboratory results. Additionally, some groups have worked to adapt these general population scores with factors specific to PLHIV (e.g. **D**ata collection on **A**dverse effects of anti-HIV **D**rugs-D:A:D), accounting for issues such as length and type of ART treatment history and renal function. These have been found to be more sensitive and specific when being evaluated in a large population of HIV positive patients [8]. However, these tests have not yet been evaluated on large SSA populations receiving ART.

As many PLHIV in SSA are now stable on ART for over a decade, with their HIV and ART exposure as well as age is increasing [9], rolling out situationally appropriate screening for CVD is becoming urgent and important. We established the Anti-retroviral Long Term (ALT) Cohort of 1000 PLHIV receiving ART for at least 10 years at the Infectious Diseases Institute (IDI), Kampala Uganda [10]. In this study we applied the most frequently used screening tools for assessing CVD risk in PLHIV from SSA populations to our cohort. We then compared the levels of agreement between the generated scores (those including lipids and non-lipids based, as well as HIV-specific and non-HIV specific) as applied to our cohort.

## Methods

### Review of tools used for cardio-vascular risk prediction by other cohorts in sub-Saharan Africa

We performed a literature search (any paper ever published on CVD risk prediction in SSA as of 31^st^/August 2018) on PubMed to identify primary articles on use of CVD risk screening in SSA including PLHIV. We included all original research articles that reported on prediction models, tools and scores that have been proposed for estimation of individual risk of any CVD that have been used in SSA including general populations (including PLHIV) or exclusively PLHIV populations. We excluded articles not written in English (or no translation available) where we were unable to access the full text through a license at our institutes. The search terms can be found in supplementary material.

### Applying cardiovascular screening tools to the ART Long Term Cohort study population

Between May 2014 and September 2015 we established the ART Long Term Cohort (ALT cohort), a prospective observational cohort of 1000 adult patients previously on ART in their consecutive 10th year of ART. The cohort has been described elsewhere [10]. Baseline and annual visits include medical history, physical examination, CD4 count, viral load, and information on ART and other co-medications information collected and entered into an electronic medical record (Integrated clinical Enterprise Application(ICEA [11]). Biological samples (packed cells, plasma and serum) were stored at enrolment and follow-up visits; full blood count, urea and electrolytes, as well as CD4, viral load and urine dipstick are done at each visit. Participants receive a blood pressure check performed by a nurse, weight measured using floor scales and BMI calculated in kgs/m^2^ calculation. Table 1 gives details on how risk factors are ascertained within the IDI cohort. Lipid tests (Total Cholesterol, LDL, HDL and triglycerides) were retrospectively performed (at Rakai Health Sciences Program laboratory) on stored serum baseline samples. Hypertension is diagnosed by having either three or more consecutive systolic and diastolic Blood Pressure (BP) measurements above 140 and 90 respectively, or self-reported, or being on antihypertensive drugs. Diabetes is evaluated from medical history, glucose in urine followed by a plasma glucose analysis > 100mg/ml. Cardio-vascular end points (CVA, MI and other e.g. valvular disease) at enrolment are self-reported.

**Table 1:** Data evaluated for CVD risk evaluation in the ALT cohort.

Risk	How evaluated	Units of measurement
Hypertension (systolic BP)	• Manual blood pressure taken by health care worker Or • Self-reported Or • On anti-hypertensive medication	>3 measurements systolic >140 or diastolic >90 or Recorded on EMR by diagnosis and medication
Body Mass Index	Height and weight at entry to cohort Weight in Kg / height^2^	Kg/m^2^
Diabetes	1) Self-reported 2) On diabetic medication 3) Diagnosed in clinic after abnormal blood glucose	EMR recorded diagnosis or medication Glucose range? (4.0–6.0 mmol/l
Total cholesterol	Roche Cobas chemistry analyzer machine	Reference range (3.5 and 5.7 mmol/L)
Triglycerides	Roche Cobas chemistry analyzer machine	Reference range (0.10–10.00 mmol/L)
Total/HDL cholesterol levels	Roche Cobas chemistry analyzer machine Or reported hyperchoestrolaemia and on medication	Reference range (0.08–3.1 mmol/L) EMR record of medication
Demographics	Self-reported	Age (years), sex
CD4 count baseline	Facs Calibur machine	Count recorded at entry into cohort
Anti-retroviral treatment exposure	From ICEA records	Drug exposure recorded on EMR

**CVD**—Cardiovascular Disease, **IDI**—Infectious Disease Institute, **BP**–Blood Pressure, **EMR**—Electronic Medical records, **ICEA**- Integrated Clinical Enterprise Application.

### Calculating absolute CVD risk scores using the CVD risk tools

Following the analysis of most commonly used tools in SSA, we used the most common tools (summarized in supplementary Table 1 in S1 File) and applied them to the ALT cohort study population enrolment visit data. These were then entered into a cross-classification table showing the number of patients in each level of risk for each tool. Where there were variance in model, we estimated the CVD risk for both the reduced (not including ART exposure data) and the full model.

### Comparison of these differing tools and resulting scores in our population

We summarised the generated scores using, medians for continuous variables, frequencies and percentages for categorical variables. We used a Prevalence Bias Adjusted Kappa (PABAK) by [12] equivalent to the weighted Brennan and Prediger [13] agreement coefficient for ordinal scale ratings to assess the level of agreement of a given pair of the CVD risk tools in cross classifying patients. A p-value of <0.05 was considered significant to assess levels of agreement. Both the crude and PABAK and 95% confidence level are presented.

We undertook the following comparisons 1) lipids vs no lipids to see if it is possible to screen without laboratory lipid evaluation 2) Inclusion or no inclusion of CVD family history. There are both 5 and 10-year prediction scores. For 5- year and 10-year predictions, we used D:A:D and ASCVD respectively as the main tools for the comparisons, as these are the scores incorporating most parameters in the prediction. The levels of agreement between two scores were assessed on the categories as per the set risk classification of the tools. The ASCVD tool only allows for 2 levels of classification (low and moderate or high or very high). So for any comparison of other scores with the ASCVD, re-classification of patient’s risk into these two categories was done. Data management, generation of scores for the different tools and the analysis was conducted using STATA Version 14.

#### Ethics

The ALT Cohort was approved by the research ethics committee of the Joint Clinical Research Centre and was also approved by the Uganda National Council for Science and Technology. The study is registered with Clinical trials.org (https://clinicaltrials.gov/) as ‘Outcomes of HIV Infected Individuals after Ten Years on Antiretroviral Treatment’ Protocol Record ST/0113/15. Our work adheres to the ‘Strengthening the Reporting of Observational Studies in Epidemiology’ (STROBE) recommendations that provide guidance on the reporting of cohort studies (described elsewhere [10]). All patients were sensitized about the study, offered inclusion and written informed consent was sought at cohort enrollment by the cohort nurse, counsellor or medical officer.

## Results

### Review of tools used for cardio-vascular risk prediction by other cohorts in sub-Saharan Africa

Table 2 shows all ten studies we found in for screening in PLHIV in SSA; seven of these were comparisons between different tools and 3 cross sectional screenings using one tool; seven studies predicted 10-year risk, one predicted 5-year risk and two had a combination of five and ten years. Four studies incorporated HIV specific risks through use of the D:A:D score; all of these were comparative studies. Only two studies incorporated World Health Organisation / International Society for Hypertension (WHO/ISH) blood tests sparing screening, all other studies required laboratory blood test analyses of lipids. The most commonly used scores were WHO/ISH CVD risk prediction charts [14], the Framingham Equation [15], the D:A:D and the Framingham Equations calibrated to the D:A:D data [16,17] and the Pooled Cohort equation for Atherosclerotic Cardiovascular Disease (ASCVD) from the American College of Cardiology/American Heart Association (ACC /AHA) [18].

**Table 2:** Published work on CVD risk scores in sub-Saharan Africa.

Title	Year	Country	Study populations	Number of patients	Scores used
Assessment of cardiovascular risk factors in people with HIV infection treated with ART in rural South Africa: A cross sectional study [19]	2015	South Africa	HIV infected patients	214	Compared 10-year risk Framingham (Lipids) equation and 5-year D:A:D equation
Cardiovascular disease risk prediction by the American College of Cardiology (ACC)/American Heart Association (AHA) Atherosclerotic Cardiovascular Disease (ASCVD) risk score among HIV-infected patients in sub-Saharan [20]	2017	Botswana	HIV infected patients	208	10- years risk. Pooled Cohorts Equation ASCVD and Framingham (LIPIDS) equation
Relationship between estimated cardiovascular disease risk and insulin resistance in a black African population living with HIV: a cross-sectional study from Cameroon [21]	2017	Cameroon	HIV infected patients	452	5-year risk by both Framingham (Lipids) and the D:A:D CVD risk equations
Dyslipidemia and cardiovascular disease risk profiles of patients attending an HIV treatment clinic in Harare, Zimbabwe [22]	2015	Zimbabwe	HIV infected patients	235	Framingham risk scores
High Prevalence of Metabolic Syndrome and Cardiovascular Disease Risk Among People with HIV on Stable ART in South western Uganda [23]	2016	Uganda	HIV infected patients	271	Framingham risk score
Risk factors and assessment for cardiovascular disease among HIV-positive patients attending a Nigerian tertiary hospital [24]	2016	Nigeria	HIV infected patients	283	Framingham–lipid, 10 year risk & Systematic Coronary Risk Evaluation (SCORE)
The burden of hypertension, diabetes mellitus, and cardiovascular risk factors among adult Malawians in HIV care: consequences for integrated services [25]	2016	Malawi	HIV infected patients	952	Framingham CVD risk and WHO/ISH score
Distribution and performance of cardiovascular risk scores in a mixed population of HIV-infected and community-based HIV-uninfected individuals in Uganda [26]	2018	Uganda	HIV infected and Non-HIV infected people	205	FRAM-BMI, FRAM lipids, Reynolds risk score; ASCVD, D:A:D
Short-term and long-term cardiovascular risk, metabolic syndrome and HIV in Tanzania. [27]	2016	Tanzania	HIV infected and Non-HIV infected people	454	ASCVD
Metabolic disorders and cardiovascular risk in treatment-naive HIV-infected patients of sub Saharan origin starting antiretroviral: impact of westernized lifestyle [28]	2015	Côte d’Ivoire.	HIV patients starting ART	245	10 tear risk of WHO/ISH, FRAM-lipids,D:A:D,

**D:A:D—D**ata collection on **A**dverse effects of anti-HIV **D**rugs.

### Applying cardiovascular screening tools to the IDI cohort

Of the 1000 ALT cohort patients, a total of 966 patients were included in the analysis. Of the 34 that were excluded from this analysis, four were excluded for being above 75 years and 14 were missing lipid information. Sixteen had a history of CVD end points out of which 10 (62.5%) with a previous cerebro-vascular event (CVA) and 6 (37.5%) with various other cardiovascular conditions including dilated Cardiomyopathy(DCM) (n = 2), pulmonary hypertension (n = 2), valvular heart disease (n = 1), and arrhythmia (n = 1). The baseline characteristics are presented in Table 3.

**Table 3:** Descriptive summary of ALT cohort cardiovascular risk factors at enrolment (after 10 years of ART).

	**n = 966**	**%**
Sex, Female	601	62.2
Current smoker,	23	2.4
Ex-smoker	195	20.2
Diabetes	30	3.1
Hypertension	246	25.5
Ever used protease inhibitors	180	18.6
Current use of Abacavir	7	0.7
Current use of antihypertensive treatment	220	22.8
HIV RNA >400 copies/ml, n (%) ≥ 400copies/ml, n (%)	36	3.7
	**Median**	**IQR**
Age (years)	45	40–50
HDL cholesterol (mmol/L)	1.20	0.98–1.48
Total Cholesterol (mmol/L)	4.73	4.07–5.40
Body mass index (Kg/m^2)^	22.4	19.8–25.4
Duration on PI (years)	5.4	2.3–7.0
Duration on NNRTI(years)	9.6	9.4–9.8
Systolic BP(mmHg)	120	110–130
CD4 count at cohort 2 enrollment cell/μL	505	362–684
CD4 count (Pre-ART) cell/μL (range)	81	26–155

**ALT** cohort—ART Long Term Cohort.

Of the 966 patients, 601 (62.2%) were women, the median age was 45 (IQR: 40–54) years, 246 (25.5%) had a diagnosis of hypertension, of which 220 (89.4%) were on anti-hypertensive treatment: 30 (3.1%) had diabetes. Smoking history included 195 (20.2%) ex-smokers and 23 (2.4%), current smokers. For ART history, 180 (18.6%) had ever used Protease Inhibitors (PIs) for a median (IQR) duration of 5.4 (2.3–6.8) years. Only six (0.6%) were current users of abacavir. For those still on first line the median (IQR) duration on Nucleoside Reverse Transcriptase Inhibitors (NRTIs) was 9.6 (9.4–9.8) years. The median (IQR) CD4 count was 505 (362–684) cells/μL; the median (IQR) HDL cholesterol and total cholesterol was 1.20 (IQR 0.98–1.48) and 4.73 (IQR 4.08–5.40) mmol/L respectively, median (IQR) body mass index was 22.4 (19.8–25.4) Kg/m^2^, and the systolic BP 120 (IQR 110–130) mmHg.

Table 4 and supplementary Table 2 in S1 File show the application of all the main tools to the ALT cohort. The 10 year generated scores showed both higher risk and wider range (percentage at risk of getting CVD over 10-year event across all scores has a median of 1.7–3.8%) than the 5 year generated scores (percentage at risk of an event over 5 years across all scores has a median 1.1–1.4%). The ASCVD and Framingham lipids scores show higher percentage of participants at high risk, compared to Framingham BMI. Framingham lipids 5 years also showed a higher percentage of PLHIV at CVD high risk compared to both D:A:D scores.

**Table 4:** Median(IQR) Percentage Scores of cardio-vascular risk over 5 or 10 years, applied to the ALT cohort.

Score	Prediction period (years)	Median(IQR)	Rating	n(%)	Median(IQR)
ASCVD–(%)	10	2.5(0.70–5.4)	low	816(84.5)	1.7(0.5–3.8).
			High/high	150(15.5)	11.9(9.4–16.2)
Fram-BMI– 10 year risk (%)	10	1.7(0.8–3.1)	low	919(95.1)	1.6(0.7–2.9)
			Moderate	36(3.7)	12.5(11.2–14.0)
			High	11(1.1)	32.9(21.7–36.3)
Fram Lipids-10 year risk	10	3.8(2.1–7.7)	low	801(82.9)	3.1(1.9–5.1)
			Moderate	117(12.1)	13.0(11.3–15.1)
			High	48(5.0)	27.0(22.6–36.7)
Fram-lipids-5 year risk(%)	5	1.4(0.9–2.5)	low	310(32.1)	0.7(0.5–0.9)
			Moderate	568(58.8)	1.8(1.3–2.6)
			High	88(9.1)	6.7(5.6–8.3)
D:A:D reduced (%)	5	1.1(0.2–1.9)	low	460(47.6)	0.6(0.4–0.8)
			Moderate	472(48.9)	1.8(1.3–2.6)
			High/very high	33(3.4)	6.8(6.0–8.6)
D:A:D with full model(%)	5	1.2(0.7–2.1)	low	404(42.0)	0.6(0.5–0.9)
			Moderate	526(54.7)	1.8(1.3–2.5)
			High/very high	31(3.2)	7.0(5.9–8.7)
WHO with cholesterol	10	NA	low	946(97.9)	NA
			Moderate	13(1.3)	
			High/ Very high	7(0.7)	

ASCVD (Atherosclerotic Cardiovascular Disease) only allows for 2 levels of classification—low or moderate/ high/ very high.

BMI—Body Mass Index.

WHO—World Health Organization.

D:A:D**—D**ata collection on **A**dverse effects of anti-HIV **D**rugs.

Fram-BMI Framingham CVD risk prediction score that used BMI instead of lipids.

Fram lipids Framingham CVD risk prediction score that used lipids instead of BMI.

**Fig 1:**
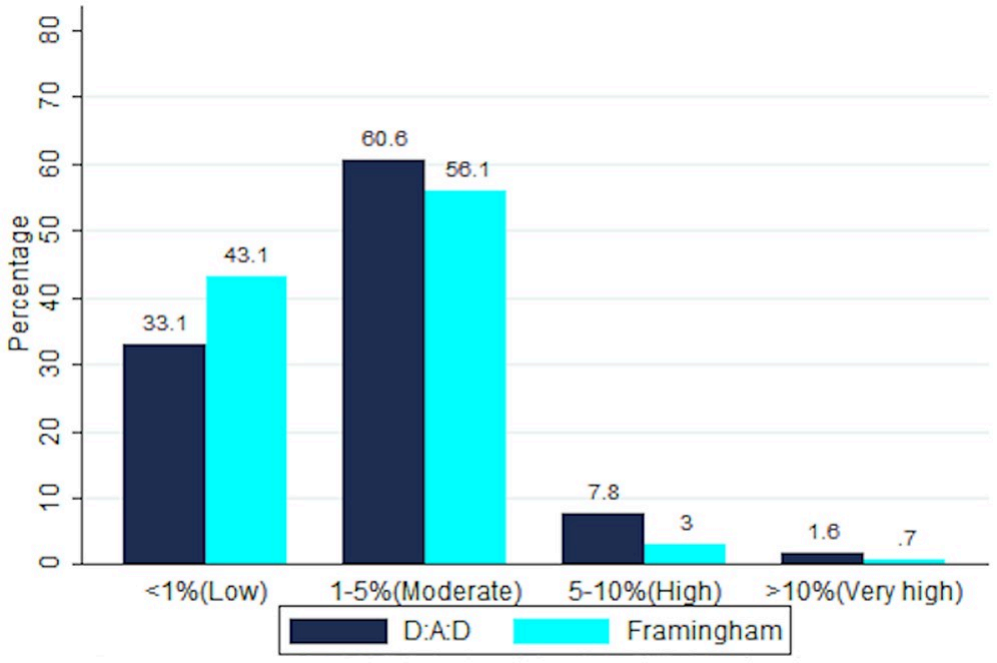
Classification of ALT cohort participants in CVD risk categories by Framingham (5 year) and full DAD 5-year risk equations.

Fig 1 and Table 6 show a head-to-head comparison of full model D:A:D, (which is specific to PLHIV) and Framingham 5 year. Of the 966, both models categorized 251 individuals as low risk, 409 as moderate risk, 16 as high risk and 6 as very high risk (supplementary Table 2 in S1 File). The median (IQR) predicated 5-year CVD risk of the D:A:D and the Framingham was 1.2% (0.7–2.1) and 1.4% (0.9–2.5) respectively (Table 4). A comparison of the 5-year risk scores gave a level of agreement of 90.1% (PABAK = 0.76; 95% CI 0.74–0.79; p value<0.001).

#### Comparison of score results across the cohort

Cross-classification was mapped in a contingency table (supplementary Table 2 in S1 File) with nine comparisons for 10-year prediction scores and two for 5-year prediction scores. The PABAK was used to determine level of agreement between scores that included lipid analysis and scores with no lipid analysis as presented in Tables 5 and 6. The paired comparisons yielded moderate to substantial level of agreement for all the comparisons (Table 5). Table 6 compares HIV specific versus non-HIV specific 5-year scores, which yielded moderate to substantial level of agreement for all the paired comparisons.

**Table 5:** Level of agreement between CVD risk equations including blood testing for lipids and no lipid testing as applied to the ALT cohort.

	Predicted 10-year CVD risk scores		
Score	Fram-Lipids	ASCVD	WHO with cholesterol
**WHO with no cholesterol**	90.1% 0.78(0.75–0.82),<0.001	86.5% 0.73(0.690.77),<0.001)	99.0% 0.98(0.97–0.99), <0.001
**Fram-BMI**	90.0% 0.78(0.74–0.81), <0.001	86.0% 0.72(0.67–0.76), <0.001	95.7%% 0.90(0.88–0.93),<0.001

Key Crude level of agreement.

PBAK (95% Confidence Interval of PBAK).

**Table 6:** Level of agreement between HIV specific CVD risk equations and non-HIV specific equations as applied to the ALT.

	Fram-Lipids
**Full D:A:D model**	90.1% 0.76(0.74–0.79), <0.001
**Reduced D:A:D model**	89.4% 0.75(0.72–0.77), <0.001

Key Crude level of agreement.

PBAK 95% Confidence Interval of PABAK).

## Discussion

In this study, we have applied a variety of screening tools to a cohort of PLHIV in Uganda who have been on ART for at least 10 years. We used screening tools used by researchers around the continent and we found no statistical difference in the risk scores generated when comparing the different tools applied to our population. Overall, the Ugandan ALT cohort has a median score of 1.1–1.4% risk of a CVD event over 5 years and 1.7–2.5% risk of a CVD event over 10 years. The strength of this study is that we undertook a review of all the tools used prior to the analysis and applied multiple tools to the ALT cohort; this yielded very similar results. This risk is similar to other cohorts in Africa [28]. There are an increasing number of publications related to screening for cardiovascular disease in PLHIV in SSA [19–28], however there are multiple screening tools used and there is no consensus about which is the best to use. In summary, there are 3 main variations between the most commonly used tools. These are 1) requirement for lipid results 2) length of prediction time (risk over 5 years and risk of 10 years) 3) HIV specific criteria and adjustments.

In resource limited settings, the major challenges to screening include capacity (training and available time) of health care workers, and availability of laboratory testing mainly driven by financial constraints. Particularly, lipid analysis specifically is costly (approximately US$11) and not routinely available in most health centres. Therefore, we evaluated the screening tools comparing non-lipid dependent tools and lipid dependent tools (Table 6). Despite the higher estimated number of people with high risk if lipids were used, there was a high level of agreement in risk classification of patients seen between generated scores including and not including lipid profiles. This is reassuring for those who are screening for cardiovascular disease without lipid availability, and either WHO or Fram-BMI would be suitable and straightforward to use. These both result in a 10-year prediction of CVD risk.

We also compared HIV specific tests (D:A:D full and D:A:D restricted—Table 6), which give 5 year predictions and these were in statistical agreement with the Framingham- Lipid 5 year score. D:A:D categorized more cohort participants as high risk (Fig 1), which is unsurprising as it is adjusting for extra risk associated with ART use. In a SSA setting, due to widespread use of public health monitoring tools for HIV, ART history is likely to be widely available, therefore a full D:A:D may be possible. Unfortunately, both 5-year tools required lipid analysis and we did not find tools that adjusted for HIV and ART related factors, without need for a laboratory lipid analysis. It would be helpful to have a resource limited D:A:D score that removes need for lipids, but does factor in ART history. Additionally, development and deployment low cost point of care lipid evaluation for low income settings should be an urgent research priority.

A limitation of this study is that it is a cross sectional analysis of the tools, and we have not yet reached a follow up point to assess validity of the tools. Therefore, we have only been able to assess agreement between tools in use in this setting. Another limitation is a challenge in comparing 5 year and 10 year scores, which causes a little confusion in comparisons, but we have tried to address this by providing clarity in the tables. Another limitation is that the ALT cohort was missing information on family history, which is key in some of the tools. This is not specific to the ALT cohort, as generally in SSA death certification, cause of death assessments, and post mortems are not common, so it is common that relatives do not know the cause of death in their family members. Therefore, inclusion of this question in screening tools may lead to underestimated or overestimated scores.

So far in the published literature on CVD screening in PLHIV in SSA, these screening tools have been used for cross sectional analysis and have not yet been used for long-term follow up of PLHIV. This is due to the relative paucity of long-term non-HIV related morbidity and mortality data as compared to Europe and North America, due to the more recent availability of ART in Africa. The ALT cohort, as well as others in the region, is approaching a five-year follow up period and we will assess the accuracy of the scores against our 5-year outcomes. However, as our cohort and others described are reasonably small, a pooled analysis of this data would provide more statistical power.

## Conclusion

Given the burden of HIV in Uganda, if 3.4% of the PLHIV population are at a high risk of a CVD event, this will equate to around 48,000cardiovascular events in PLHIV in Uganda over the next 5 years. As this risk is modifiable with lifestyle advice and treatment of risk factors such as diabetes and hypertension, screening and patient education seems a sensible addition to routine HIV care. Our analysis suggests that the most common screening tools globally may provide useful information in our environment. However, generally in our resource-limited setting, use of cardiovascular screening tools is severely restricted by availability of lipid test results and family history information. Whilst we are hesitant to suggest development of new screening tools to add to an already crowded field, it would seem prudent to consider an HIV related adjustment of some of the more simple tools. We would encourage low cost lipid testing development or adjustment of D:A:D to remove need inclusion of laboratory lipid testing or other tools to include ART history. We suggest that some of these simple adaptions may be a pragmatic approach to improving sensitivity and ease of use in resource-limited settings. When our ALT cohort reaches five-year follow up, we will start to evaluate these screening tools for our population.

## Supporting information

S1 File (DOCX)
