# Supplementary material for: Comparison of different cardiovascular risk tools used in HIV patient cohorts in sub-Saharan Africa; do we need to include laboratory tests?
Source: PLoS One. 2021 Jan 28;16(1):e0243552. doi: 10.1371/journal.pone.0243552 (PMC7842918; doi:10.1371/journal.pone.0243552)
Supplement: S1 File — (DOCX) [file pone.0243552.s001.docx]

**Supplementary material**

**Search terms for paper review**

(((((((Cardiovascular risk prediction OR Cardiovascular prediction OR Cardiovascular risk score OR performance of cardiovascular risk scores)) OR (CVD risk prediction OR CVD prediction)) OR (cardiovascular risk assessment OR CVD risk assessment)) OR (cardiovascular risk models OR cardiovascular risk scores OR cardiovascular risk equations OR cardiovascular risk algorithms OR cardiovascular risk tools OR performance of cardiovascular risk scores)) AND ("D:A:D" OR "DAD" OR data collection on adverse effects of anti-HIV drugs)) OR (ASCVD OR American Society For Hypertension OR Atherosclerotic Cardiovascular Disease Or American College Of Cardiology And American Heart Association score) OR WHO/ISH OR Framingham OR FRS-BMI OR FRAM BMI AND (Africa OR Sub-Saharan Africa) AND (prediction OR assessment OR estimation OR estimating OR performance))

**Table 1: Requirements for CVD prediction using different models and how these were applied these in the analyses**

| **Prediction model** | **VARIBLES** | | | | | | | | | | | | | | | | | | | |  |
| --- | --- | --- | --- | --- | --- | --- | --- | --- | --- | --- | --- | --- | --- | --- | --- | --- | --- | --- | --- | --- | --- |
|  | Years of prediction | Gender-(male/female) | Age(years) | *Diabetes history, Yes/No* | *Systolic BP (mmHg)* | *Total Cholesterol(mmol/* *mg/dL) – (LT)* | *BMI- Body Mass Index(kgs/m^3^);* | *Current smoking status-Yes/No* | *Previous smoking status- Yes/No* | *HDL chol-esterol-(mmol/* *mg/dL) – (LT)* | *Duration on PI (years)* | *Duration on NRTI(years)* | *Current CD4 count(cells/µL)* | *CVD Family History- Yes/No* | *Currently on abacavir- Yes/No* | *Ethnic back-ground/race* | *Statin use- Yes/No* | *Aspirin use - Yes/No* | *LDL Cholesterol(mmol/* *mg/dL); -( LT)* | *On Hyper-tension treatment- Yes/No* | *Application of these models in the analyses* |
| WHO/ISH risk prediction chart AFRE- with Cholesterol(L) | 10 | ✔ | ✔ | ✔ | ✔ | ✔ |  | ✔ |  |  |  |  |  |  |  |  |  |  |  |  | We used sub regional classification for AFR E where blood cholesterol can or cannot be measured. The 10-year risk of CVD was classified as <10%; moderate, 10–19.9%; high, 20–29.9%; and very high risk, ≥30%(11) |
| WHO/ISH risk prediction chart AFRE- without Cholesterol(NL) | 10 | ✔ | ✔ | ✔ | ✔ |  |  | ✔ |  |  |  |  |  |  |  |  |  |  |  |  |  |
| FRAM-lipids (L) | 10 | ✔ | ✔ | ✔ | ✔ | ✔ |  | ✔ |  | ✔ |  |  |  |  |  |  |  |  |  |  | We calculated a 10-year risk using both the Framingham equation with Lipids (FRAM-Lipids, Primary Model) and without lipids (FRAM-BMI,Simple Office-based non-laboratory model). ((7). The 10-year risk of CVD was classified <10% – low risk; 10%-<20% – moderate risk; ≥20% – high risk(17, 18). We also estimated a 5-year risk of CVD using an algorithm for calculating the calibrated Framingham risk. The 5-year predicted risk for each individual’s was categorised as one of the following categories: <1%-very low, 1–5%-moderate, 5–10%-high and >10%-very high (12, 13). (See algorithms in supplementary material). |
| FRAM-BMI (NL) | 10 | ✔ | ✔ | ✔ | ✔ |  | ✔ | ✔ |  |  |  |  |  |  |  |  |  |  |  |  |  |
| FRAM-lipids (D:A:D Primary Model)(L) | 5 | ✔ | ✔ | ✔ | ✔ | ✔ |  | ✔ |  | ✔ |  |  |  |  |  |  |  |  |  | ✔ | We calculated a 5-year risk of developing CVD using **D**ata collection on **A**dverse effects of anti-HIV **D**rugs (D:A:D) equation(20). We estimated the CVD risk for both the reduced (not including ART exposure data) and the full model. We also estimated a 5-year risk of CVD using an algorithm for calculating the calibrated Framingham risk. The 5-year predicted risk for each individual’s was categorised as one of the following categories: <1%-very low, 1–5%-moderate, 5–10%-high and >10%-very high (20, 21). |
| FULL D:A:D (F) CVD 5 year risk score(L) | 5 | ✔ | ✔ | ✔ | ✔ | ✔ |  | ✔ | ✔ | ✔ | ✔ | ✔ | ✔ | ✔ | ✔ |  |  |  |  |  |  |
| Restricted D:A:D (R) CVD 5 year risk score(L) | 5 | ✔ | ✔ | ✔ | ✔ | ✔ |  | ✔ | ✔ | ✔ |  |  | ✔ | ✔ |  |  |  |  |  |  |  |
| ASCVD(L) | 10 | ✔ | ✔ | ✔ | ✔ | ✔ |  | ✔ |  | ✔ |  |  |  |  |  | ✔ | OP | OP | ✔ | ✔ | We estimated a 10-year CVD risk using the instructions given by (13, 15) in the guidelines on the Assessment of Cardiovascular Risk. |

**Supplementary Table 2: Cross classification of participants by different CVD risk score models**

| **Predicted 10-year CVD Risk** | | | | | | | | | | | | | | | |
| --- | --- | --- | --- | --- | --- | --- | --- | --- | --- | --- | --- | --- | --- | --- | --- |
| **WHO with no cholesterol-10 year** | **WHO with cholesterol-10 year** | | | |  | **Fram-Lipids-**  **10 year** | **Fram-BMI-10 year** | | | | **Fram-Lipids-**  **10 year** | **WHO with cholesterol-10 year** | | | |
|  | <10%  (Low) | 10<20%  (Moderate) | 20<30%  (High) | ≥30%  (Very high) | Total |  | <10%  (Low) | 10<20%  (Moderate) | >=20%  High/very high | Total |  | <10%  Low | 10<20%  Moderate | >=20%  High/very high) | Total |
| <10% | 935 | 8 | 2 | 1 | 946 | <10% | 787 | 13 | 1 | 801 | <10% | 779 | 2 | 0 | 801 |
| 10<20% | 0 | 7 | 4 | 2 | 13 | 10<20% | 92 | 21 | 4 | 117 | 10<20% | 105 | 8 | 4 | 117 |
| 20<30% | 0 | 2 | 0 | 1 | 3 | >=20% | 40 | 2 | 6 | 48 | >=20% | 31 | 7 | 10 | 48 |
| ≥30% | 0 | 0 | 0 | 4 | 4 |  |  |  |  |  |  |  |  |  |  |
| Total | 935 | 17 | 6 | 8 | 966 | Total | 919 | 36 | 11 | 966 | Total | 935 | 14 | 13 | 966 |
| **ASCVD- 10 year risk** | **Fram-Lipids- 10 year risk** | | |  |  | **Fram-BMI-10 year risk** | **WHO with no cholesterol- 10 year risk** | | |  | **Fram-Lipids-10 year** | **WHO with no cholesterol-10 year** | | | |
|  | <10%  (Low) | ≥10%(moderate/high/  very high) | | Total |  |  | <10%  (low) | 10<20%(  Moderate ) | >=20%  High/very | Total |  | <10%  Low | 10<20%  Moderate | >=20%  High/very high) | Total |
| <7.5(Low risk) | 784 | 32 | | 816 |  | <10%  Low | 902 | 10 | 7 | 919 | <10%(Low) | 800 | 1 | 0 | 801 |
| >=7.5%(High) | 17 | 133 | | 150 |  | 10-<20 | 35 | 1 | 0 | 36 | 10<20% | 109 | 6 | 2 | 117 |
|  |  |  | |  |  | >20 | 9 | 2 | 0 | 11 | (>=20% | 37 | 6 | 5 | 48 |
| Total | 801 | 165 | | 966 |  | Total | 946 | 13 | 7 | 966 | Total | 946 | 13 | 7 | 966 |
| **ASCVD- 10 year risk** | **WHO with cholesterol-10 year risk** | | | | |  | **WHO with no cholesterol- 10 year risk** | | | | **ASCVD- 10 year risk** | **Fram-BMI-10 year risk** | | |  |
|  | <10%  (Low) | ≥10%(moderate/high/  very high) | | Total | | **ASCVD- 10 year risk** | <10% | ≥10%(moderate/high/  very high) | | Total |  | <10%  (Low) | ≥10%(moderate/high/  very high) | | Total |
| <7.5(Low risk) | 814 | 2 | | 816 | | <7.5(Low) | 816 | 0 | | 816 | <7.5(Low risk) | 799 | 17 | | 816 |
| >=7.5%(High) | 121 | 29 | | 150 | | >=7.5%(high) | 130 | 20 | | 150 | >=7.5%(High) | 120 | 30 | | 150 |
| Total | 935 | 31 | | 966 | | Total | 946 | 20 | | 966 | Total | 919 | 47 | | 966 |
| **Predicted 5- Year CVD risk** | | | | | | | | | | | | | | | |
| **Framingham**  **5-year risk** | **D:A:D full 5-year risk** | | | | | **Framingham**  **5-year risk** | **Reduced D:A:D 5-year risk** | | | | | | |  |  |
|  | <1%  (low risk) | 1-5%  (moderate) | 5-10  (High) | >10%  (very high) | Total |  | <1%  (low risk) | 1-5%  (moderate) | 5-10  (High) | >10%  (very high) | | Total | |  |  |
| <1% | 251 | 153 | 0 | 0 | 404 | <1% | 266 | 194 | 0 | 0 | | 460 | |  |  |
| 1-5% (moderate risk) | 59 | 409 | 55 | 3 | 526 | 1-5% (moderate risk) | 44 | 370 | 55 | 3  3 | | 472 | |  |  |
| 5-10% | 0 | 6 | 16 | 6 | 28 | 5-10% | 0 | 4 | 16 | **6** | | 26 | |  |  |
| >10% | 0 | 0 | 1 | 6 | 7 | >10% | 0 | 0 | 1 | 6 | | 7 | |  |  |
| Total | 310 | 568 | 72 | 15 | 965 | Total | 310 | 568 | 72 | 15 | | 965 | |  |  |

**Table 3: Interpretation of Kappa**

| Kappa | Agreement |
| --- | --- |
| < 0 | Less than Chance agreement |
| 0.01–0.20 | Slight agreement |
| 0.21– 0.40 | Fair agreement |
| 0.41–0.60 | Moderate agreement |
| 0.61–0.80 | Substantial agreement |
| 0.81–0.99 | Almost perfect agreement |

**Note: Interpretation Same for PBAK**

**Algorithms and Graphs**

1. **The D:A:D model and the Framingham model calibrated to the D:A:D DATA**

<http://login.research4life.org/tacsgr0journals_sagepub_com/doi/suppl/10.1177/2047487315579291/suppl_file/suppl-appendix.pdf>

<http://login.research4life.org/tacsgr0journals_sagepub_com/doi/pdf/10.1177/2047487315579291>

<https://www.chip.dk/Tools-Standards/Clinical-risk-scores>

Calculating the risk scores:

**Full D:A:D model**

First calculate the predicted covariate score, normalised to the mean covariate values:

Xb = ( 3.0904*ln(age) + 0.313892*male + 0.6749906*diabetes + 0.3143615*famgp +

0.8088174*currsmk +0.2129574*exsmk + 0.9481744*ln(chol) - 0.500635*ln(hdl) +

1.523599*ln(syst) - 0.1188231*ln2(cd4) + 0.3838412*nonabc + 0.0466745*cumpi +

0.0278108*cumnuc )

-

(3.0904*ln(40.412) + 0.313892*0.7442 + 0.6749906*0.0308 + 0.3143615*0.0840 +

0.8088174*0.5217 + 0.2129574*0.1670 + 0.9481744*ln(5.001) - 0.500635*ln(1.214)

+ 1.523599*ln(123.651) - 0.1188231*ln2(482.099) +0.3838412*0.1365 +

0.0466745*1.718 + 0.0278108*3.313 )

The predicted five-year risk is estimated using the Cox five-year survival at the mean values of predictors,

S(5)=0.9853, using

Predicted risk = 1 - 0.9853^exp(Xb)^

# Reduced D:A:D model

Xb = ( 3.1777*ln(age) + 0.343856*male + 0.7311945*diabetes + 0.329772*famgp +

0.8157995*currsmk + 0.2394822*exsmk + 1.0925460*ln(chol) - 0.5194359*ln(hdl) +

1.517874*ln(syst) - 0.1137227*ln2(cd4) )

-

( 3.1777*ln(40.412) + 0.343856*0.7442 + 0.7311945*0.0308 + 0.3297720*0.0840 +

0.8157995*0.5217 + 0.2394822*0.1670 + 1.0925460*ln(5.001) - 0.519436*ln(1.214)

+1.517874*ln(123.651) 0.1137227*ln2(482.099) )

Predicted risk again = 1 - 0.9853^exp(Xb)^

# *Framingham Model*

The Framingham model calibrated to the D:A:D data is calculated in the same manner, but for men and women separately.

**For women**

Xb = ( 2.32888*ln(age) + 0.69154*diabetes + 0.52873*currsmk + 1.20904*ln(chol) –

0.70833*ln(hdl) + (1-bptrt)*2.76157*ln(syst) + bptrt*2.82263*ln(syst) )

-

(2.32888*ln(37.3763) + 0.69154*0.0227 + 0.52873*0.4325 + 1.20904*ln(192.2218) -

0.70833*ln(54.63029) +0.9467*2.76157*ln(117.875) + 0.0533*2.82263*ln(134.683) )

The predicted five-year risk for women is then estimated using the Cox five-year survival in women at the mean values of predictors, S(5)=0.9883, using

Predicted risk = 1 – 0.9883^exp(Xb)^

For men

Xb = ( 3.06117*ln(age) + 0.57367*diabetes + 0.65451*currsmk + 1.12370*ln(chol) –

0.93263*ln(hdl) + (1-bptrt)*1.93303*ln(syst) + bptrt*1.99881*ln(syst) )

-

( 3.06117*ln(41.4552) + 0.57367*0.0335 + 0.65451*0.5524 + 1.12370*ln(193.8152)

0.93263*ln(44.27783) +0.9389*1.93303*ln(124.579) + 0.0611*1.99881*ln(136.839)

Predicted risk again = 1 - 0.9840^exp(Xb)^

Where: famgp = family history of CVD

nonabc = currently on abacavir

cumpi = cumulative exposure to protease inhibitors

cumnuc = cumulative exposure to nucleoside reverse transcriptase inhibitors

bptrt = currently receiving blood pressure lowering treatment (1=yes, 0=no)

ln = log (base e), ln2 = log (base 2)

Technical note:

The full D:A:D model is valid for cumulative NRTI exposure up to about 8-10 years, and PI exposure to around 5-6 years. Extrapolating beyond these exposures without recalibrating will lead to over estimates of CVD risk. For patients who are highly exposed to antiretroviral treatment, use of the reduced D:A:D model is recommended.

1. **Framingham 10- year risk CVD Model(FRAM-BMI and FRAM-lipids)**

<https://www.framinghamheartstudy.org/fhs-risk-functions/cardiovascular-disease-10-year-risk/>

## **ASCVD (Atherosclerotic Cardiovascular Disease (ASCVD) event)**

<https://www.ahajournals.org/doi/abs/10.1161/01.cir.0000437741.48606.98>

1. **WHO/ISH Risk prediction charts for 14 WHO epidemiological sub-regions (AFR e and AFR D)**

<http://ish-world.com/downloads/activities/colour_charts_24_Aug_07.pdf>
